# Exploring the transformative role of 3D printing in advancing medical education in Africa: a review

**DOI:** 10.1097/MS9.0000000000001195

**Published:** 2023-08-14

**Authors:** Gbolahan Olatunji, Osadebamwen W. Osaghae, Nicholas Aderinto

**Affiliations:** a Department of Medicine and Surgery, University of Ilorin, Ilorin; b Department of Medicine and Surgery, Ladoke Akintola University of Technology, Ogbomoso, Nigeria; c Johns Hopkins Bloomberg School of Public Health, Maryland, USA

**Keywords:** Africa, 3D printing, medical education

## Abstract

With the increasing demand for quality healthcare and the scarcity of resources, medical education in Africa faces numerous challenges. Traditional teaching methods often need help to adequately prepare medical students for the complex and diverse healthcare scenarios they will encounter in practice. 3D printing technology holds significant promise in addressing these challenges by providing innovative solutions for medical education. This review examines the various applications of 3D printing in medical education, focusing on its potential to enhance anatomy education, surgical training and medical device development. It explores how 3D printing can offer realistic and customisable anatomical models, enabling students to understand human anatomy better and improve their surgical skills through realistic simulations. Furthermore, this paper discusses the potential of 3D printing in developing low-cost medical devices, prosthetics and surgical instruments, which can significantly benefit resource-limited settings in Africa. It explores the concept of distributed manufacturing, where 3D printing can decentralise the production of essential medical equipment, reducing reliance on external suppliers and improving access to healthcare. The review also highlights the challenges and limitations associated with implementing 3D printing in medical education in Africa, such as limited infrastructure, high costs and the need for specialised training. However, it presents successful initiatives and collaborations that have overcome these obstacles, demonstrating the feasibility and potential impact of integrating 3D printing into medical education in Africa.

## Introduction

Highlights

- There is a critical need for advanced medical education in Africa to address healthcare challenges and improve patient outcomes.
- The shortage of resources and the limited access to learning models hinder effective medical training.
- 3D printing can help create precise anatomical models, surgical tools and medical devices, revolutionising the learning experience for African medical students.

Medical education has a long history of embracing innovation and incorporating new technologies to enhance learning and improve patient care^1^. In recent years, 3D printing technology has emerged as a transformative tool with immense medical potential^2^. Its ability to create detailed and anatomically accurate physical models has revolutionised how medical students learn and prepare for clinical practice^1^. In Africa, medical education faces unique challenges due to the diverse healthcare landscape and resource constraints^3^. 3D printing technology presents a promising solution to address these challenges and reshape medical education in Africa^4^. By leveraging 3D printing, medical educators can create anatomically accurate models that closely mimic real human structures, providing an invaluable opportunity for students and trainees to visualise and manipulate complex anatomical systems offering a level of realism and interactivity that traditional educational resources cannot replicate. Additionally, 3D printing enables the production of patient-specific anatomical models derived from medical imaging data, enabling surgeons to anticipate challenges, optimise surgical strategies and improve patient outcomes^5^.

Furthermore, this technology can be particularly impactful in Africa, where access to advanced medical imaging and surgical resources is often limited^5^. By democratising access to patient-specific models, 3D printing can bridge the gap in healthcare training and enable healthcare professionals to provide more precise and tailored care. However, some challenges need to be addressed for widespread adoption, such as the cost of equipment and materials, technical expertise in 3D printing and the establishment of quality standards. This narrative review will critically evaluate the current literature on 3D printing in medical education, focusing on its applications, effectiveness and limitations. It will also explore successful case studies from Africa and other regions, highlighting innovative approaches and best practices. By harnessing the potential of 3D printing, Africa can pave the way for innovative educational approaches, bridge the gap in healthcare training and contribute to improved healthcare outcomes for its population.

## Methodology

A comprehensive literature search was conducted to identify pertinent articles addressing the utilisation of 3D printing in medical education. Multiple databases, including PubMed, Google Scholar and Medline, were systematically searched using the following keywords: ʻ3D printingʼ, ʻmedical educationʼ, and ʻAfricaʼ. The search strategy combined these terms using Boolean operators (AND, OR, NOT) to refine the results. The initial database searches yielded a total of 120 articles. The reference lists of the identified articles were also reviewed to ensure a comprehensive review, leading to the acquisition of additional articles. In total, 136 articles were obtained for detailed assessment.

A predefined set of inclusion criteria was applied to determine the final selection of articles for the review. These criteria included a focus on 3D printing in medical education, publication in peer-reviewed journals, no limitation on publication year and the requirement for articles to be in English. Following the application of these criteria, a thorough screening process was undertaken. Articles were assessed based on their relevance to the topic, peer-reviewed status, language and alignment with the study’s objectives. Articles that did not meet these criteria were excluded from further consideration. As a result of this rigorous screening process, 31 articles were deemed relevant and subsequently included in the review. These articles provided valuable insights and information regarding the transformative role of 3D printing in advancing medical education in Africa.

To provide a visual representation of the article selection process, a table (see Table 1) is presented, illustrating the step-by-step progression from the initial number of retrieved articles to the final inclusion of 31 relevant articles for the review. The table demonstrates the systematic approach to selecting the articles for this study.

**Table 1:** Article selection process for reviewing the utilisation of 3D printing in medical education in Africa.

Stage	Description	Number of articles
Initial search	Database search using keywords ʼ 3D printingʼ, ʼ medical educationʼ, and ʼ Africaʼ	120
Reference review	Review of reference lists of identified articles	+16
Total articles	Total number of articles acquired for detailed assessment	136
Screening	Application of predefined inclusion criteria	
- Relevance	Articles aligned with the focus on 3D printing in medical education	45
- Peer-reviewed	Articles published in peer-reviewed journals	38
- Language	Articles in English	36
Final selection	Articles meeting all inclusion criteria	31

## Current state of medical education in Africa

In Africa, the landscape of medical education has undergone significant transformations. Former European colonies established medical schools and postindependence governments prioritised medical education. Sub-Saharan Africa experienced a notable increase in medical schools, particularly from 1990 to 2009^6^. However, a 2015 study identified six African nations without medical schools, while 20 countries had only one^7^. The medical curriculum in many African schools follows a 6-year system similar to British undergraduate medical training, distinguishing it from other Western countries.

In contrast, the United States and the United Kingdom offer more flexibility, allowing students to pursue integrated research or management programs known as intercalated medical programs. Some students even pursue dual degrees, expanding their educational horizons^8^. For instance, in Nigeria, medical graduates receive their medical degrees after completing their undergraduate years and then undertake a mandatory 1-year internship at accredited hospitals. Following this, doctors can apply for residency training programs^9^. These variations in medical curricula across African countries reflect the diverse approaches to medical education, influenced by historical legacies, regional needs and healthcare system structures. While some African medical schools have embraced innovative approaches, such as integrating research or offering dual degree programs, others have maintained a more traditional structure, emphasising clinical training and specialisation after completing undergraduate studies.

In Sub-Saharan Africa (SSA), medical education has embraced innovative approaches to address unique challenges. These approaches include community-based education and service (COBES), problem-based learning (PBL), assessment and evaluation techniques, technology integration, and retention strategies. COBES offers advantages such as lower attrition rates and effective functioning in rural communities^10^. PBL enhances learning strategies, collaboration and communication skills^10^. However, implementing PBL in SSA faces high costs, inadequate materials and faculty shortages. COVID-19 has further impacted medical training institutions in Africa. PPE shortages posed safety concerns and lockdowns disrupted in-person training, leading to the adoption of remote learning^11^. Exam cancellations and delays affected student progression^11^.

African medical education institutions have embraced technological solutions to overcome challenges. Technology integration, including online platforms, virtual simulations and telemedicine, enables continued learning during the pandemic^11^. Strategies ensure PPE availability and appropriate use for safety. Retention efforts include rural exposure programs, financial incentives and career development in underserved areas^11^. Ongoing investment in infrastructure, faculty development and resources is needed to sustain advancements. Collaboration between institutions globally fosters knowledge exchange and best practices.

The pandemic’s impact extends to the financial aspects of African medical education. Government-owned and private universities face reduced revenues from budget cuts and decreased school fees^12^. Teaching hospitals experience decreased revenues due to fewer patients seeking care^12^. Addressing ongoing challenges in medical education requires innovation. Shortages of healthcare professionals hinder service delivery. Changing disease burdens necessitate an evolving approach focusing on prevention and management. Contextual differences require tailored curricula. Embracing technologies like 3D printing is crucial for promoting innovation in medical education.

## Potential benefits of 3D printing in medical education

3D printing holds immense potential to revolutionise medical education, providing innovative and transformative approaches to learning about the human body, surgical procedures and medical devices. Table 2 summarises the key benefits of 3D printing in medical education. By utilising 3D-printed anatomical models, students can better understand complex anatomical structures, facilitating engaging and hands-on learning experiences. These models allow for enhanced visualisation and interaction with anatomical structures, fostering a more comprehensive understanding of medical concepts and procedures. Moreover, 3D printing enables the creation of patient-specific models for preoperative planning and simulation. This advancement enhances surgical training and contributes to improved patient outcomes. By integrating 3D printing into medical education, African institutions can overcome traditional barriers to learning, such as limited access to cadavers and other educational resources. In addition to its educational benefits, 3D printing can support the development of customised medical devices and prosthetics, addressing the specific needs of patients in resource-limited settings. This technology empowers healthcare professionals to create tailored solutions that improve patient care and outcomes.

**Table 2:** Comparative analysis of traditional medical education methods vs. 3D printing-based medical education.

Aspect	Traditional medical education	3D printing-based medical education
Teaching tools	Cadavers, anatomical models, textbooks	3D-printed anatomical models, virtual simulations
Cost considerations	Expensive cadavers, limited availability	Affordable 3D printers, materials, and software
Accessibility	Limited access to cadavers and specialized centres	Widely accessible with portable 3D printers and digital resources
Level of learner engagement	Limited hands-on experience, passive learning	Active learning, hands-on experience with 3D models
Learning outcomes	Good understanding of anatomy, limited surgical practice	Improved spatial understanding, enhanced surgical skills
Interactivity	Limited interaction with complex anatomical structures	Interactive exploration and dissection of 3D models
Customization	Limited options for customization	Customised anatomical models based on patient data
Procedural training	Limited opportunities for repetitive practice	Realistic surgical simulations for procedural training
Innovation potential	Limited scope for innovation in teaching methods	Facilitates innovative teaching methods and research
Future applications	Limited integration of emerging technologies	Potential integration with augmented reality and virtual reality

Although the adoption of 3D printing in medical education in Africa may face challenges, such as limited access to technology and the need for appropriate training, there are opportunities for collaboration and investment. Partnerships between academic institutions, healthcare providers and technology companies can facilitate the acquisition of 3D printers, training programs and curriculum development. Governments also play a crucial role by providing funding, research support and regulatory oversight to ensure the safe and effective implementation of 3D printing in medical education. These collective efforts can drive the integration of 3D printing into medical curricula, empowering African institutions to deliver high-quality education and advance healthcare outcomes.

A study by McMenamin *et al*.^13^ explored the practical applications of 3D printing in medical education, specifically focusing on teaching human pathological specimens to medical students. The researchers highlighted a shift in medical schools towards online teaching of anatomical pathology using 2D images, which has resulted in a decreased emphasis on teaching medical anatomical pathology. However, the introduction of 3D printing in pathology education offers several advantages.

One of the key benefits is the reduction in maintenance time and the elimination of health and safety concerns associated with handling pathology specimens in fluid-filled glass containers. By incorporating 3D-printed replicas, the teaching of clinical pathology for medical students and pathology trainees can be enhanced, making it applicable in various educational settings. Acknowledging that using 3D-printed copies of human pathology specimens has certain limitations is important. However, addressing these limitations, such as ensuring high-quality input for producing high-quality output, is essential for effective implementation. Another example showcasing the benefits of 3D printing in medical education comes from a collaborative project between Macquarie University and Western Sydney University in Australia^14^. The project aimed to create highly precise 3D prints of human bones from the Macquarie University Skeletal Collection. These 3D prints were then utilised in anatomy laboratories, allowing students to handle and examine the specimens physically.

These examples demonstrate the tangible advantages of integrating 3D printing into medical education. By utilising 3D-printed replicas, educators can offer practical and interactive learning experiences, addressing the limitations of traditional teaching methods. 3D printing in medical education can revolutionise how students learn and engage with complex anatomical structures and pathological specimens, ultimately enhancing their understanding and preparedness for clinical practice.

3D models in medical education have been shown to enhance the comprehension of complex medical concepts significantly. In a study focusing on paediatric straddle injury repair, 3D paediatric vulvar models were utilised to simulate surgical experiences and proved effective teaching tools. Participants’ knowledge scores improved by an impressive 85% after utilising the models alongside a didactic session^15^. In another study, 3D-printed injection moulds were employed to construct gynaecologic surgical simulation models using polyvinyl alcohol. Attending surgeons considered the models realistic (83.3%) and believed they accurately represented the critical steps of a myomectomy (87.5%). Residents expressed that practising on the models would better prepare them for a myomectomy (87.5%), and attending surgeons showed a willingness to grant residents more operative autonomy if they had completed the simulation (71.4%)^16^.

In a unique approach, researchers developed 3D puzzle pieces of eyeball models to assist ophthalmology students in comprehending the intricate structures of the eye^17^. Similarly, researchers created a scale model representing the musculature of the lower limb and posterior compartment using the BodyParts3D dataset. The effectiveness of the 3D-printed model was evaluated in comparison to the traditional dissection room method. The 3D-printed model group achieved an average score of 50% in a Limb Model Assessment, indicating its potential as an invaluable tool in anatomy education. The authors anticipate that 3D printing will become increasingly essential in anatomy education, especially as the range of printable materials expands and the technology becomes more cost-effective^18^.

3D printing technology has also become indispensable in various medical fields. In cardiovascular interventions like percutaneous mitral valve repair and transcatheter aortic valve replacement, 3D printing is crucial in preprocedural planning and preparation, enhancing surgical planning and patient care^19–21^. In Maxillofacial Surgery, 3D printing technology is increasingly used for dental implant surgery and mandibular reconstruction^22^. Adopting 3D printing in medical education and clinical practice offers promising opportunities to improve learning outcomes, surgical training and patient care across various disciplines. As the technology continues to evolve and new materials are developed, its impact is expected to expand further, benefiting educators and healthcare professionals.

From an economic standpoint, 3D printing offers a cost-effective alternative, particularly beneficial for regions with relatively weaker economies, such as Africa. A study by Chen *et al*.^23^ demonstrated the cost-effectiveness of 3D printing technology in spinal deformity surgery. This showcases the potential for cost savings and improved accessibility to medical interventions.

In another study by Frithioff *et al*., the cost-effectiveness of low-cost 3D-printed temporal bone models for training in mastoidectomy procedures was demonstrated. These models, created using entry-level 3D printing technology and a heat-resistant filament, proved to be valuable introductions to mastoidectomy and supplements to cadaveric temporal bones. The study highlights the potential for institutions to create cost-effective temporal bone models with various anatomical variations, enhancing training opportunities^24^.

3D printing in medical education and surgical training can help overcome financial barriers, allowing institutions to provide realistic and accessible training models and tools at a fraction of the cost compared to traditional methods. This cost-effectiveness allows institutions in resource-limited settings, such as Africa, to enhance medical education and training without incurring significant financial burdens.

## 3D printing in medical education in Africa

The utilisation of 3D printing in medical care and education in Africa has yet to be explored, with limited available data on its specific applications in the region. A review focused on 3D printing in neurosurgical education revealed a need for more representation from the African continent^25^. However, there are notable instances where 3D printing has been successfully deployed in Africa. One such example is the Department of Agricultural and Bio-Systems Engineering (DABE) at Makerere University’s College of Agricultural and Environmental Sciences (CAES), which introduced innovative technologies in August 2020 in response to the challenges posed by the COVID-19 pandemic. Among these technologies were thermal imaging, 3D printing of biodegradable face shields and producing components for the Bulamu Ventilator. The 3D-printed biodegradable face shields, designed with antimicrobial properties and easy cleanability, proved invaluable in the battle against the virus^26^.

Meanwhile, South Africa is advancing medical device innovation through the Medical Device Additive Manufacturing Demonstrator Project (MedAdd) in Bloemfontein^27^. This groundbreaking initiative harnesses the power of additive manufacturing, or 3D printing, to produce customised medical devices. With an impressive production output of over 2000 unique medical products, MedAdd aims to address the country’s disease burden by providing locally developed and contextually relevant medical devices, diagnostics and active pharmaceutical ingredients^28^. The MedAdd project bridges the innovation gap for medical device companies and improves the quality of life for individuals with disabilities, showcasing the transformative potential of 3D printing in South Africa.

While comprehensive data on the applications of 3D printing in Africa’s medical landscape is still limited, these examples highlight the technology’s potential to tackle healthcare challenges and drive innovation across the continent. Further exploration and documentation of 3D printing’s applications in Africa hold promise for enhancing medical care and education, ultimately leading to improved health outcomes and advancements in medical research.

## Integrating 3D printing technology into the medical education framework in Africa

Integrating 3D printing technology into the medical education framework in Africa holds substantial promise for transforming healthcare training and delivery. However, successful implementation necessitates meticulous planning and thoughtful consideration. To begin, a comprehensive needs assessment is crucial in identifying the specific areas within medical education where the integration of 3D printing can yield the greatest impact. Engaging educators, medical professionals and students allow for a comprehensive understanding of their requirements and challenges, guiding the focus areas for effective implementation.

Stakeholder engagement is paramount during the needs assessment phase as it fosters a collaborative approach incorporating diverse perspectives. By actively involving educators, medical professionals and students, the integration efforts can align with their specific needs, resulting in a more tailored and effective integration of 3D printing into the medical education landscape.

Simultaneously, evaluating African medical institutions’ available resources and infrastructure is critical. This evaluation examines the accessibility of 3D printing equipment, materials and software. If additional resources are required, seeking partnerships or funding opportunities becomes imperative to acquire the necessary assets or establish collaborations with existing 3D printing facilities. Following the needs assessment, developing a comprehensive strategic plan is essential to guide the integration process. This plan outlines the objectives, strategies and actionable steps necessary for successful implementation. Key considerations include curriculum integration, faculty training, infrastructure development, ethical aspects, sustainability and evaluation.

Curriculum integration focuses on seamlessly incorporating 3D printing activities into the existing curriculum to optimise learning outcomes. This may involve creating new modules, adapting current courses, or establishing dedicated workshops that enable students to acquire knowledge of 3D printing principles and their application within medical education contexts. Simultaneously, faculty training is vital in ensuring educators possess the necessary proficiency to effectively harness 3D printing technology and guide students in its application. Providing training programs, workshops and access to educational resources enhances faculty members’ understanding of 3D printing principles, software tools, design considerations and troubleshooting techniques. Encouraging faculty members to explore innovative teaching methodologies that seamlessly integrate 3D printing further augments their pedagogical prowess.

Facilitating collaborations and partnerships with industry experts, research institutions and experienced medical education establishments expedites the integration process. These partnerships provide access to expertise, shared resources and opportunities for collaborative projects, fostering knowledge exchange, developing best practices and aligning with the latest advancements in 3D printing. In addition, ethical considerations are paramount in integrating 3D printing technology. Developing comprehensive guidelines and protocols ensures the responsible and ethical utilisation of 3D printing technology in medical education. These guidelines encompass crucial aspects, such as patient privacy, informed consent, data security and intellectual property concerns. Educating faculty members, students and other stakeholders about these ethical considerations is pivotal to ensuring adherence to professional standards.

Similarly, sustainability planning assumes a critical role in successful implementation. Establishing long-term strategies for the maintenance, funding and expansion of 3D printing infrastructure is necessary. This includes budgeting for material costs, regular equipment maintenance and training new staff members. Seeking funding opportunities, exploring collaborations with local and international organisations and developing sustainable business models contribute to the long-term viability of 3D printing initiatives in medical education.

Evaluation and feedback mechanisms are indispensable for assessing the effectiveness of integrating 3D printing technology. Ongoing evaluation enables the measurement of learning outcomes, the identification of areas for improvement and the validation of the impact on healthcare training and delivery. Soliciting feedback from students, educators and healthcare professionals facilitates the identification of strengths and weaknesses in 3D printing activities, enabling continuous improvement and refinement of the integration process.

## Future directions and recommendations

In recent years, 3D printing technology has witnessed increased accessibility and affordability in developed countries. However, integrating 3D printing technology into medical education in Africa requires a significant financial investment. The cost of integrating 3D printing technology encompasses several factors. First and foremost, there is an initial investment in acquiring the necessary equipment, including 3D printers, scanners and computer-aided design software. The cost of these devices varies depending on the desired specifications and quality, ranging from a few hundred dollars to several thousand dollars. Procuring equipment of sufficient quality is crucial to ensure accurate and reliable output. In addition to equipment costs, the expenditure on materials used for 3D printing, such as filaments or resins, must be considered. Material selection depends on the specific applications and requirements of the medical education programme. While a wide range of materials is available at different prices, specialised or medical-grade materials may be more expensive. Balancing cost-effectiveness with the desired quality and properties of the printed objects is essential.

Training and professional development for faculty and staff implementing 3D printing technology contribute to the overall cost. Workshops, courses, or consultations may be necessary to equip educators with the skills and knowledge to effectively utilise 3D printing in medical education. These training programs often involve additional expenses, such as registration fees, travel costs and the engagement of expert trainers or consultants. Ensuring that faculty members are proficient in the technical aspects of 3D printing and its pedagogical applications is crucial for successful integration.

Maintenance and operational costs constitute another significant aspect to consider. 3D printers require regular maintenance, calibration and potential repairs like any technological equipment. Allocating resources for these maintenance activities is essential to ensure the continued functionality and longevity of the equipment. Additionally, ongoing expenses such as electricity, software updates and material replenishment should be factored into the budget. Despite the initial investment and associated costs, it is important to recognise the long-term benefits that can outweigh the financial implications.

Conducting a thorough cost-benefit analysis is essential to evaluate the return on investment and the long-term sustainability of integrating 3D printing technology into medical education. This analysis should encompass a comprehensive assessment of the potential improvements in learning outcomes, enhanced practical skills for students and the overall impact on healthcare delivery in Africa. By systematically evaluating the costs against the anticipated benefits, educational institutions can make informed decisions and allocate resources effectively.

**Figure 1:**
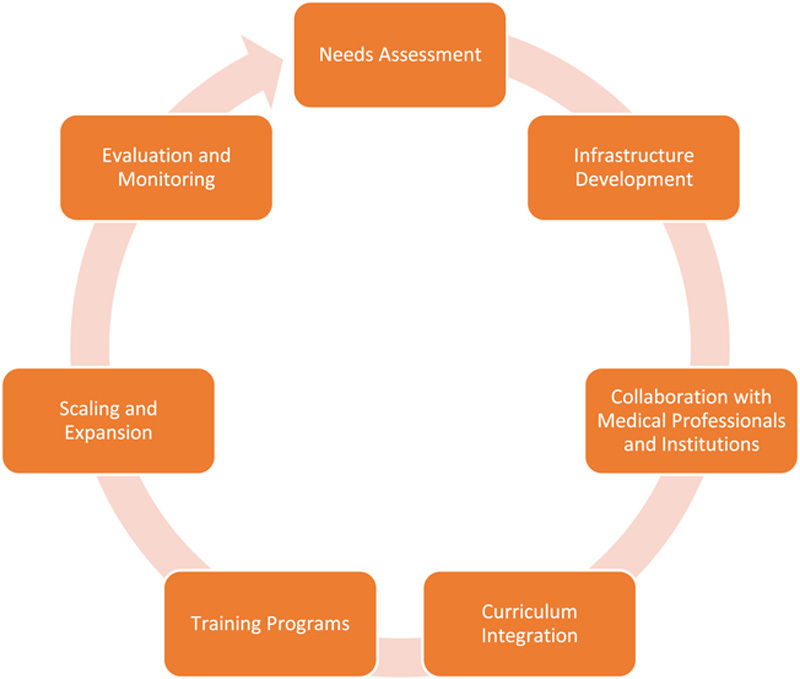
Implementation strategy for integrating 3D printing in medical education in Africa.

Integrating 3D printing technology into medical education in Africa has significant barriers, including limited access to technology, a lack of funding, insufficient knowledge and skills and a standardised curriculum^29^ (Fig. 1). These challenges pose obstacles to effectively utilising 3D printing and hinder medical students’ ability to learn about its applications in medicine. However, despite these barriers, there are opportunities to implement 3D printing in medical education in Africa, and collaborative efforts play a vital role in driving progress.

To advance medical education through 3D printing, academic institutions and medical professionals must join forces. By fostering collaboration, universities, medical schools and healthcare professionals can exchange knowledge, research and develop effective educational approaches leveraging 3D printing technology. Sharing expertise and experiences can help address the knowledge and skills gap, ensuring that educators and students are well-equipped to utilise 3D printing effectively^30^.

Government support is also critical in overcoming barriers to adopting 3D printing in medical education. Government agencies can play a pivotal role by providing the necessary funding, supporting research and development initiatives and ensuring regulatory oversight to guarantee the safety and efficacy of 3D-printed models^31^. By actively supporting 3D printing in medical education, governments can help alleviate the financial burdens associated with acquiring the necessary technology. Initiatives and grants from the government can play a significant role in addressing medical schools and institutions’ financial constraints, enabling them to invest in 3D printing technology and training programs.

Furthermore, establishing a standardised curriculum for integrating 3D printing into medical education is essential. By developing guidelines and educational frameworks, medical education institutions can ensure that students receive comprehensive and standardised training in 3D printing technology. Collaboration between educational institutions, professional associations and industry experts can contribute to developing such a curriculum, considering African medical education’s specific needs and contexts.

Partnerships with international organisations present a valuable avenue for advancing medical education through 3D printing in Africa. These partnerships offer access to expertise, resources and funding opportunities, contributing to capacity building and knowledge sharing. Collaborating with international organisations can significantly enhance the implementation of 3D printing in medical education across the continent. By leveraging the knowledge and resources of these organisations, African institutions can accelerate their adoption of 3D printing technology and integrate it effectively into their curricula.

Collaborations between companies also play a crucial role in achieving widespread access to 3D printing in medical education and healthcare in Africa. By partnering with 3D printing companies, medical schools can access affordable and high-quality printing solutions. For example, the partnership between MedTech3D and Axial3D aims to bring accessible 3D printing capabilities to hospitals in South Africa. Such collaborations facilitate sharing resources, expertise and innovative solutions, ultimately benefiting African medical education and healthcare.

Advocacy campaigns and efforts are essential in raising awareness about the advantages of 3D printing in medical education. By advocating for integrating 3D printing technology, these campaigns can foster collaboration and investment in the field. Increased awareness and support will lead to a greater understanding of the potential benefits of 3D printing, encouraging stakeholders to embrace this technology and allocate resources to its implementation. To address the limited access to 3D printing technology, African medical schools can establish partnerships with 3D printing companies and organisations. Through these collaborations, medical schools can access 3D printers and receive training on their proper usage. Additionally, exploring the concept of shared 3D printing facilities can help reduce the cost of acquiring and maintaining printers, making them more accessible to medical education institutions.

Overcoming the financial challenges of implementing 3D printing technology in medical education requires seeking external funding. Medical schools in Africa can actively pursue government grants, seek support from private organisations and engage with philanthropists invested in advancing healthcare education. Crowdfunding platforms can also be utilised to raise funds for 3D printing technology, enabling medical schools to acquire the necessary resources.

Further research is needed to advance the understanding of the transformative role of 3D printing in advancing medical education in Africa. One area of future research involves assessing the educational outcomes of integrating 3D printing technology into medical education. Conducting systematic evaluations can help determine the impact of 3D-printed anatomical models on student learning, skill acquisition and clinical performance. Comparative studies can be conducted to assess the effectiveness of 3D printing compared to traditional educational approaches, such as cadaver dissection or computer-based simulations. Additionally, research focusing on customising and personalising 3D-printed models can greatly enhance their impact on medical education. Exploring patient-specific anatomical models derived from medical imaging data can provide valuable surgical planning, simulation and training opportunities. Investigating the feasibility and effectiveness of creating region-specific anatomical models that reflect Africa’s unique healthcare challenges and population characteristics can further enhance the relevance and applicability of 3D printing technology in Africa.

Furthermore, future research should explore the long-term impact and sustainability of incorporating 3D printing in medical education. This includes evaluating the retention of knowledge and skills gained through 3D printing activities and assessing the scalability and feasibility of implementing 3D printing programs in resource-limited settings.

## Conclusion

Integrating 3D printing in medical education in Africa holds immense promise for driving advancements in the field. Despite challenges, Africa can overcome obstacles to equip healthcare professionals with enhanced skills and knowledge, improving patient care. Collaboration, investment and government support are crucial. Partnerships with international organisations and companies facilitate access to resources and expertise. Advocacy campaigns raise awareness and encourage resource allocation. Seeking external funding and establishing shared 3D printing facilities address financial constraints. By embracing 3D printing, Africa can position itself at the forefront of medical education and shape the future of healthcare in the region.

## Ethical approval

Ethical approval is not applicable for this review.

## Consent

Informed consent is not applicable for this review.

## Sources of funding

No funding was received for this study.

## Author contribution

G.O. and N.A.: conceptualization. All authors contributed in writing of the article.

## Conflicts of interest disclosure

All authors declare no conflicts of interest.

## Research registration unique identifying number (UIN)

1. Name of the registry: not applicable.
2. Unique identifying number or registration ID: not applicable.
3. Hyperlink to your specific registration (must be publicly accessible and will be checked): not applicable.

## Guarantor

Nicholas Aderinto.

## Provenance and peer review

Not commissioned, externally peer-reviewed.

## Data availability statement

Data sharing is not applicable to this article.
